# Effects of cortical distance on the Ebbinghaus and Delboeuf illusions

**DOI:** 10.1177/03010066231175014

**Published:** 2023-06-19

**Authors:** Poutasi W. B. Urale, Dietrich Samuel Schwarzkopf

**Affiliations:** 1415 School of Optometry & Vision Science, The University of Auckland, New Zealand

**Keywords:** neural mechanisms, perception, crowding, eccentricity, Ebbinghaus illusion, Delboeuf illusion, size perception

## Abstract

The Ebbinghaus and Delboeuf illusions affect the perceived size of a target circle depending on the size and proximity of circular inducers or a ring. Converging evidence suggests that these illusions are driven by interactions between contours mediated by their cortical distance in primary visual cortex. We tested the effect of cortical distance on these illusions using two methods: First, we manipulated retinal distance between target and inducers in a two-interval forced choice design, finding that targets appeared larger with a closer surround. Next, we predicted that targets presented peripherally should appear larger due to cortical magnification. Hence, we tested the illusion strength when positioning the stimuli at various eccentricities, with results supporting this hypothesis. We calculated estimated cortical distances between illusion elements in each experiment and used these estimates to compare the relationship between cortical distance and illusion strength across our experiments. In a final experiment, we modified the Delboeuf illusion to test whether the influence of the inducers/annuli in this illusion is influenced by an inhibitory surround. We found evidence that an additional outer ring makes targets appear smaller compared to a single-ring condition, suggesting that near and distal contours have antagonistic effects on perceived target size.

The Ebbinghaus illusion (see Figure 1) has bamboozled our visual systems for over a century (Ebbinghaus, 1902; Titchener, 1905). Yet, despite a mountain of research on this illusion, the neural mechanisms underlying it remain poorly understood. Filling this lacuna is crucial for understanding how the brain determines visual object size, which is itself an unresolved question (Schwarzkopf, 2015).

**Figure 1.**
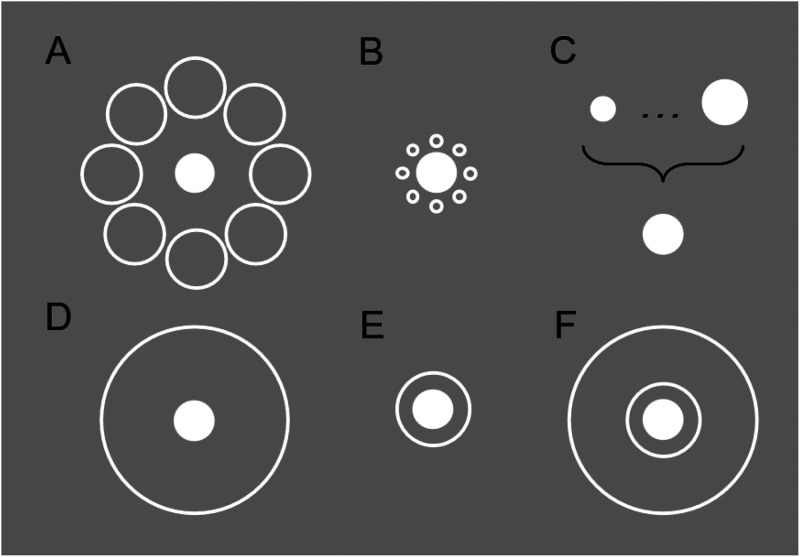
Key stimuli. (A-B) The Ebbinghaus illusion. Most observers will perceive the white filled-in circle in B (small inducers) as larger than that in A (large inducers). (C) The test targets Experiments 1, 2, 3a, and 3b varies according to a staircase procedure. (D-E) The Delboeuf illusion. Most observers will perceive the white filled-in circle in E (close ring) as larger than that in D (far ring). (F) Novel two-ring Delboeuf stimulus used in Experiment 3a, featuring near and far annuli.

## Theories of the Ebbinghaus Illusion

Several theories have attempted to explain the Ebbinghaus illusion. Most illustrations of this illusion show an apparent “size-contrast” effect, where the center circle (target) surrounded by small inducers appears larger, while large inducers make the target appear smaller (e.g., Massaro & Anderson, 1970, 1971; Obonai, 1954). Many authors have thus described the illusion in terms of this size-contrast mechanism (Aglioti et al., 1995; Haffenden et al., 2001; Yamazaki et al., 2010). However, Todorović and Jovanović (2018) point out that size-contrast is descriptive rather than explanatory and offers an incomplete account for the illusion. They argue that size contrast is nebulously defined, and that there is no explanation for why there is a size-contrast effect instead of an assimilation effect, as found for other visual illusions such as the tilt illusion (Clifford, 2014).

Part of this objection stems from the observation that geometrical features other than inducer size also modulate the strength of the Ebbinghaus illusion. One such factor is object-level similarity between targets and inducers, which make the illusion stronger (Coren & Miller, 1974; Massaro & Anderson, 1971; Rose & Bressan, 2002). The illusion also depends on the amount of empty space between inducers, with a more complete ring of inducers around the periphery of the target strengthening the illusion (Girgus et al., 1972; Massaro & Anderson, 1970; Roberts et al., 2005). Roberts et al. (2005) investigated the role of completeness by directly comparing the Ebbinghaus illusion to the Delboeuf illusion (see Figure 1), another size-perception illusion that uses a circular ring that surrounds the target stimulus instead of multiple circular inducers (Delboeuf, 1865; Evans, 1995). They showed that an Ebbinghaus configuration composed of small inducers that formed a complete ring yield about the same illusory effect as a Delboeuf configuration, and that both configurations yielded stronger effects than Ebbinghaus configurations with less complete inducer annuli. Lastly, Ebbinghaus illusion strength changes with the distance between the target and inducers (target-inducer distance). Massaro and Anderson (1971) found that point of subjective equality (PSE) decreased with greater target-inducer distances, with the farthest distances failing to affect the perceived size of the target at all. Other work (Girgus et al., 1972; Jaeger, 1978; Roberts et al., 2005; Weintraub, 1979) also showed that target-inducer distance modulates the illusion, but with an unexpected reversal of the effect of small inducers in some configurations. That is, a sufficiently large distance between the target and small inducers causes the target to appear smaller, not larger, compared to control. Similarly, Roberts et al. (2005) found that even when controlling for completeness, increasing the target-inducer distance reversed the effect of small inducers, making the target appear smaller rather than larger. In contrast, large inducers always elicited a perceived shrinkage of the target, and this effect only became stronger with inducer-target distance. These findings demonstrate that to describe the Ebbinghaus illusion as an example of “size-contrast” is an oversimplification.

Contour-based accounts (Jaeger, 1978; Jaeger & Long, 2007; Jaeger & Lorden, 1980; Sherman & Chouinard, 2016; Todorović & Jovanović, 2018; Weintraub, 1979; Weintraub & Schneck, 1986) are explanations based on interactions between the low-level contours that make up a stimulus. On the whole, these theories account better for the experimental evidence. Biphasic contour-interaction theory (BCIT) is one such account (Roberts et al., 2005; Sherman & Chouinard, 2016; Weintraub & Schneck, 1986). In contrast to the mid-level size comparison mechanism needed for a size-contrast account, the premise of BCIT explains the illusion in terms of low-level representations of contours: Nearby contours are attracted, whereas distant contours repel each other, hence the effect is “biphasic.” Roberts and colleagues’ (2005) findings are consistent with this theory, with their data showing a tendency for Ebbinghaus inducers to make the target appear smaller as target-inducer distance is increased. They found a similar effect with the Delboeuf illusion. Relatedly, Sherman and Chouinard (2016) showed a correlation between the Delboeuf illusion and Ebbinghaus illusion. They argue this is incompatible with a size-contrast account because the ring in the Delboeuf illusion is always larger than the target. Todorović and Jovanović (2018) addressed the size-contrast theory more directly by using a novel stimulus. They found that increasing the number of small inducers in an Ebbinghaus configuration can counterintuitively eliminate the illusion if the target is embedded in a grid of inducers (also see Jaeger & Klahs, 2015). Their finding disputes a size-contrast account that predicts more inducers to amplify the size contrast between the inducers and the target, while supporting a BCIT-based explanation, which posits that attractive and repellent effects of contours located at varying distances from the target cancel out.

Nevertheless, BCIT does not offer a complete explanation of the Ebbinghaus illusion. BCIT cannot explain the effect of similarity between inducers and targets when the distribution of near and far contours are controlled for (Coren & Miller, 1974; Deni & Brigner, 1997). As others have noted, there are often multiple contributing factors to visual illusions (Coren & Girgus, 1978), and it is possible that the Ebbinghaus illusion may represent the outcome of several distinct processes along the visual stream. Importantly, Rose and Bressan (2002) replicated the similarity effect and further showed that illusion magnitude was boosted only when both inducers and targets were circles or triangles, but not hexagons or irregular angular shapes. This disputes size contrast theories, as well as any contour-interaction account that is based on the sum of Euclidean distances between contours. Considering this, contour-interaction seems to be a necessary but insufficient factor in the Ebbingaus illusion. As Rose and Bressan and others (Coren & Girgus, 1978; Schwarzkopf, 2015) have pointed out, the complete explanation of the Ebbinghaus illusion may incorporate non-linear effects arising from top-down feedback, or even multiple contributing mechanisms.

## Neural Correlates of the Ebbinghaus Illusion

Converging evidence suggests that the effect of inducers on perceived size of the target is mediated by processes located in V1. Illusion magnitude was reduced—but not abolished—when inducers and target were shown to separate eyes (Song et al., 2011); indicative of a cortical mechanism in V1 where there are still many monocular neurons, although this cannot rule out a contribution from higher visual areas. Additionally, Schwarzkopf et al. (2011) used functional magnetic resonance imaging (fMRI) and retinotopic mapping to show that functional primary visual cortex (V1) surface area can predict Ebbinghaus PSE. V1's selectivity for local contrast edges makes it a likely candidate site for mediating low-level interactions as posited by the contour-interaction account. They used the classical Ebbinghaus illusion, where observers judged the difference in target size between a large-inducer and small-inducer configuration. In a follow-up study, Schwarzkopf and Rees (2013) also found a correlation between V1 area and the PSEs for large and small inducers tested separately. Both Ebbinghaus configurations made the target appear relatively larger in individuals with small V1s. The authors surmised this may indicate the effect of local circuits within V1, which are contingent on cortical distance. This could represent an attenuation of the effects of these circuits at greater distances or because of the time taken by those signals to propagate. Moreover, while large inducers reliably made the target appear smaller, small inducers made the target appear larger for some and smaller for other observers depending on their V1 surface area. Relatedly, Moutsiana et al. (2016) found that the expansive effect of the Delboeuf illusion is enhanced when it is encoded by larger population receptive fields (pRFs) in V1 (Harvey & Dumoulin, 2011). This was true both within observers with variation of pRF size across the visual field, and between individuals with different pRF sizes. While not able to explain the repulsive effect between contours, these results and those of Schwarzkopf et al. (2011) can be conceptualized as indicative of an antagonistic center-surround field of local interactions that defines a gradient of modulation based on cortical distance (Schwarzkopf, 2015). When target-inducer distance is small, there is an attractive effect, and the target appears larger. Conversely, when the distance is large the repulsive effect dominates, and the target appears smaller. In between is a point of equilibrium where inducers would have neither an attractive nor repulsive effect. The sign change of the illusion with small inducers across observers is consistent with this theory.

## In the Current Work

While Schwarzkopf and Rees (2013) and prior work (Schwarzkopf et al., 2011) has shown evidence that the Ebbinghaus illusion depends on between-subject differences in cortical topography, the present work looks at the effect of varying cortical target-inducer distance *within* individuals. As such, we manipulate cortical distance in two ways: by varying the retinal target-inducer distance in visual space (Experiment 1), and by varying the eccentricity of stimuli when target-inducer distance is constant (Experiment 2). If proximity of contours affects the illusion as described by Schwarzkopf and Rees (2013) then reduced cortical distance will modulate the illusion so that the target appears larger.

**Figure 2.**
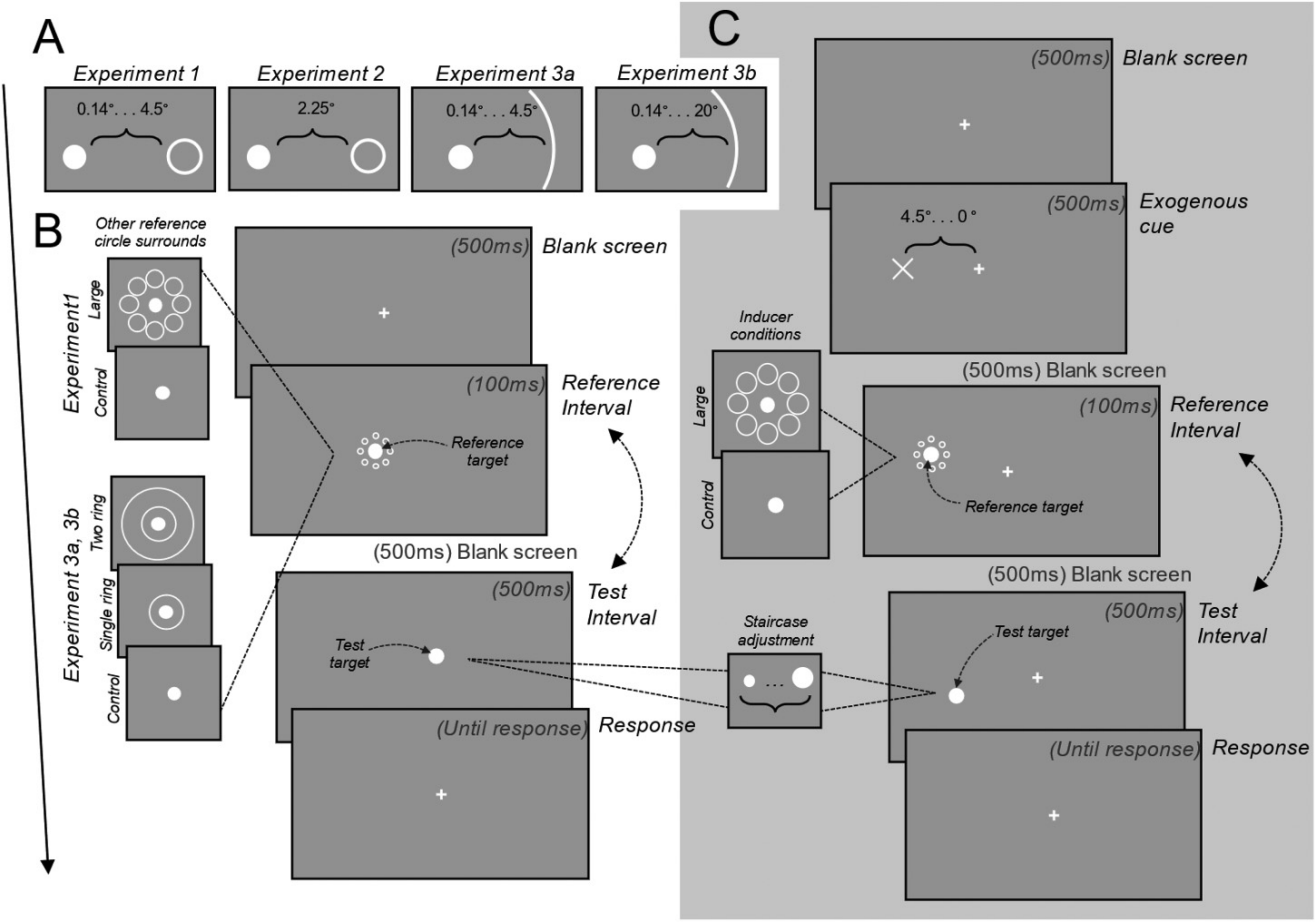
Trial procedure for Experiments 1, 2, 3a, and 3b. (A) Edge-to-edge inducer/ring distance(s) from reference target across experiments. (B) Trial sequence for Experiments 1 and 3a/3b. Observers maintained fixation on a cross before being shown reference and test intervals. The target in the test interval changed according to a staircase procedure. The order of these two intervals was counterbalanced across the experiment. Following these observers made a size comparison of targets in the two intervals. (C) Trial sequence for Experiment 2. This was like the other two experiments, except the first interval was preceded by an exogenous cue that indicated where the stimuli would appear, and gaze position was monitored using eye-tracking. Stimuli in the first and second intervals appeared above and below the horizontal meridian, respectively.

Furthermore, an account of the Ebbinghaus illusion based on cortical distance should also explain the difference in PSEs between large- and small-inducer Ebbinghaus configurations. Proponents of contour-based accounts (Sherman & Chouinard, 2016; Todorović & Jovanović, 2018; Weintraub, 1979) claim that large inducers cause a repulsive effect because they possess both near and far contours, while small inducers do not. In Experiments 3a and 3b we investigate this claim by using single- and double-ring configurations of the Delboeuf illusion. To draw further comparisons with the Ebbinghaus illusion, we also varied the retinal distance between targets and surround as in Experiment 1. If it is true that large inducers in the Ebbinghaus illusion cause repulsion because of the antagonistic effect of near and far contours, then we should observe a similar effect with the addition of a second ring in the Delboeuf illusion (Figure 2).

## Experiment 1

In Experiment 1 we varied the retinal distance between the target and the inducer. Varying the retinal distance entails changes in distances between representations of visual elements across the visual stream. This is evident in topographically organized areas such as V1 and V2, where we would expect an increase of cortical distance between representations with retinal distance. Our study here is a conceptual replication of the study by Roberts et al. (2005), who varied the distance between inducers/annuli and the target for the Ebbinghaus and Delboeuf illusions, respectively. In that study, both inducers and annuli made the target appear smaller at farther distances compared to closer distances. More recently, Knol et al. (2015) also varied the Ebbinghaus illusion along various dimensions, including target size, inducer size, and target-inducer distance, finding enlargement in cases where small- or medium-sized targets (∼0.5° and ∼1°) were displayed with inducers at short distances. In our study we used a similar manipulation with the addition of some key differences. Firstly, we included shorter target-inducer distances compared to both studies. In Roberts and colleagues’ study, the closest target-inducer distance was 1.9° for small inducers, and 2.53° for large inducers. Our study used a minimum distance of 0.14° for both inducer types. Furthermore, Roberts and colleagues limited the target-inducer distance since a closer distance would require overlap between large inducers. In our study, we allowed inducers to overlap to achieve a short target-inducer distance. The inclusion of this smallest distance tests a key hypothesis posited by Schwarzkopf and Rees (2013) who proposed that large inducers could make the target appear larger if sufficiently close. Secondly, we showed stimuli close to fixation in two temporal intervals. Most other studies testing the Ebbinghaus illusion typically use a 2-alternative forced-choice task (like ours, but where two stimuli are presented simultaneously side by side (in opposite hemifields) and are either flashed briefly (Schwarzkopf & Rees, 2013; Song et al., 2013) or remain on screen until the observer responds (Knol et al., 2015; Roberts et al., 2005; Todorović & Jovanović, 2018). Presenting stimuli in close proximity in separate intervals removes the need to split attention across the two stimulus locations and reduces the possibility of crowding effects of peripherally located stimuli.

### Materials and Methods

#### Participants

We recruited 12 observers (8 females, age range 21–50), all with normal or corrected-to-normal visual acuity. Observers provided written and informed consent and all procedures were approved by the University of Auckland Human Participants Ethics Committee (UAHPEC).

#### Experimental Setup

Stimuli were displayed on a 621 × 341 mm LCD monitor (Expt-1: Dell, S2817Q, USA; Expt-2: Samsung, U28D590D, South Korea), at a resolution of 3840 × 2160 × 8-bit resolution running 60 Hz. Monitors were linearized in software based on measurements made with a photometer (LS100, Konica Minolta, Japan). Stimuli were generated using programming environment MATLAB (version 2017B, MathWorks Inc.) and Psychtoolbox 3 (Brainard, 1997; Kleiner et al., 2007; Pelli, 1997) using customized scripts. Observers’ heads were stabilized with a chin rest.

#### Stimuli

**Figure 3.**
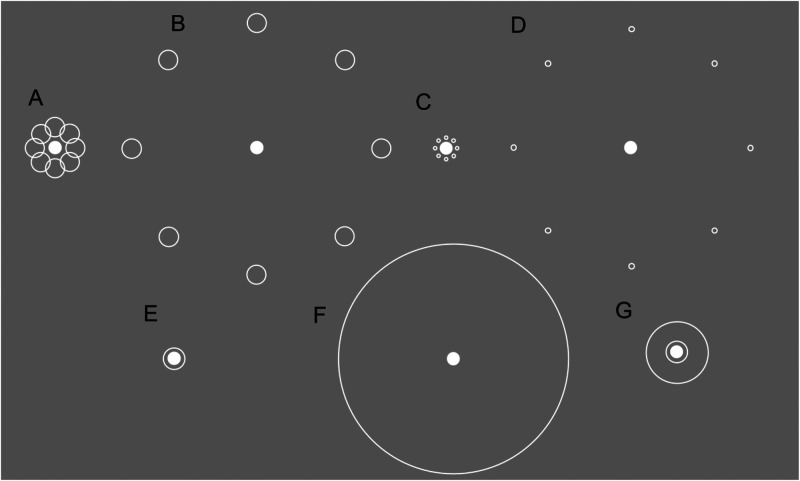
Example stimuli at various target-inducer edge-to-edge distances. (A) Large inducer Ebbinghaus configuration at 0.14° and (B) 4.5°. (C) Small inducer configuration 0.14° and (D) 4.5°. (E) Delboeuf configuration with single ring at 0.14° and (F) 4.5°. (G) Two ring Delboeuf configuration, at 0.14°.

A single trial consisted of two stimuli: a reference stimulus, consisting of a target (always 0.56° diameter) and a surround, depending on the condition of the given trial, and a test stimulus, which consisted of only a target which varied in size according to an adaptive staircase procedure (see below). Stimuli were presented on a grey (175 cd/m^2^) background. Targets were always filled, white circles (341 cd/m^2^) inducers were white outlined circles with a ∼0.08° stroke and had diameters of 0.84° and 0.2° for large and small inducers, respectively. Example stimuli can be seen in Figure 3. The edge-to-edge distance between the target and inducers (target-inducer distance) could be one of seven possible distances: 0.14°, 0.42°, 0.7°, 1.13°, 1.55°, 2.25°, and 4.5°. In addition, there was a control condition without inducers. All Ebbinghaus configurations in both experiments had eight inducers, and large inducers were allowed to overlap in conditions with very short target-inducer distances. The centers of inducers were positioned at evenly spaced radial positions relative to the target ranging from 0° to 315° in steps of 45°. Targets in each interval were shown at a horizontally offset position relative to fixation (see “Procedure” section), so in the 0.42° condition some individual inducers above and below the target overlapped the vertical meridian.

#### Procedure

Observers completed a single session lasting roughly 45 min seated in a darkened room seated at a distance of 82 cm from the screen.

Observers were given a brief verbal description of the task prior to commencement of testing. They were told to maintain fixation on a cross (0.05° × 0.05°) in the center of the monitor throughout each block. Blocks consisted of 100 trials and were separated by a rest period of at least 30 s. All stimuli were presented on a half-tone background. On each trial, stimuli were displayed sequentially with the target centered at 0.42° either left or right of fixation. The first interval always appeared just to the left of fixation, followed by the stimulus in the second interval which appeared to the right. The order of the presentation of the reference and test stimuli was decided pseudo-randomly on a per-trial basis.

At the beginning of each trial, observers saw a blank (fixation only) screen for 500 milliseconds (ms) before seeing one stimulus for ∼100 ms, followed by a blank screen again for 500 ms before the final stimulus for ∼100 ms. They were told that they would be able to respond following presentation of all stimuli. Observers pressed the left or right keyboard button to indicate whether the left or right stimulus was larger or smaller. In alternating blocks, observers were instructed to either indicate the target that appeared larger or smaller. They were also told to ignore the inducers. In case of any prior knowledge of the Ebbinghaus illusion, observers were instructed to report on their *prima facie* experience instead of what they anticipated the correct answer to be. Pressing a button to indicate their response immediately began the next trial. The ratio of the test stimulus diameter relative to the reference diameter was varied using a 1-up-1-down staircase procedure. The procedure was used to determine the PSE for each condition. With two Ebbinghaus configurations, seven target-inducer distances, and a control condition, there were a total of 15 conditions. There were two staircases for each condition, progressing in steps on a binary logarithmic scale. We chose to use a binary logarithm because it linearizes stimulus size increments in line with Weber's Law. Adjusting sizes in proportions, rather than a binary logarithmic scale as we do here, would be mathematically unsound as the non-linearity of the stimulus size ratios will theoretically skew statistical and curve fitting analyses. As an example, a stimulus half the size of the reference will have a ratio of 0.5, while a stimulus of the equivalent larger size will have a ratio of 2. The arithmetic mean of these values would be 1.25 above a ratio of 1. However, these two sizes are linearly comparable when represented as binary logarithmic units, that is, −1 (2^−1^) and 1 (2^1^), respectively. Moreover, in logarithmic units, 0 corresponds to the absence of an illusion (i.e., a size ratio of 1). Nevertheless, some readers might find it difficult to interpret logarithmic units; we therefore plot our results in linear units of degrees of visual angle but this is done purely for visualization.

On a given trial, the size of the test stimulus in degrees of visual angle was 0.56 × 2*^g^*. The staircase was varied by adjusting *g*. For each condition, one staircase began with a test diameter 0.2*g* larger than the reference target (i.e., ∼115% of the reference target diameter), and the other 0.2*g* smaller (i.e., ∼87% of the reference target diameter). The step size of the staircase varied depending on the number of reversals: 0.1*g* for trials up until the 2nd reversal, then 0.075 until the 4th reversal, followed by 0.05 until the 8th reversal, and then 0.025 for the remaining reversals (25 in total). Trials from each of the 30 staircases were randomly interleaved and discontinued after the requisite number of reversals. The experiment ended when all staircases were complete.

We calculated the PSE across conditions for each observer by fitting a cumulative Gaussian psychometric function to each condition using the weighted stimulus levels and responses from both staircases (*R*^2 ^≥ 0.98 for all fits for the present experiment and fits for psychometric functions in all subsequent experiments in this work). Assigned weighting to each data point was proportionate to the number of trials occurring at that stimulus level. All PSEs were taken as the 50% point of that function. To test the validity of our estimates we compared these values to PSEs calculated by taking the average size of the stimulus level during the last 8 reversals across both staircases for each condition, excluding values beyond twice the median absolute deviation in either direction. Using either method did not meaningfully change the pattern of results or conclusions of this manuscript. We chose a psychometric fit across all experiments as it is a more sensitive and theoretically grounded analysis.

### Results and Discussion

**Figure 4.**
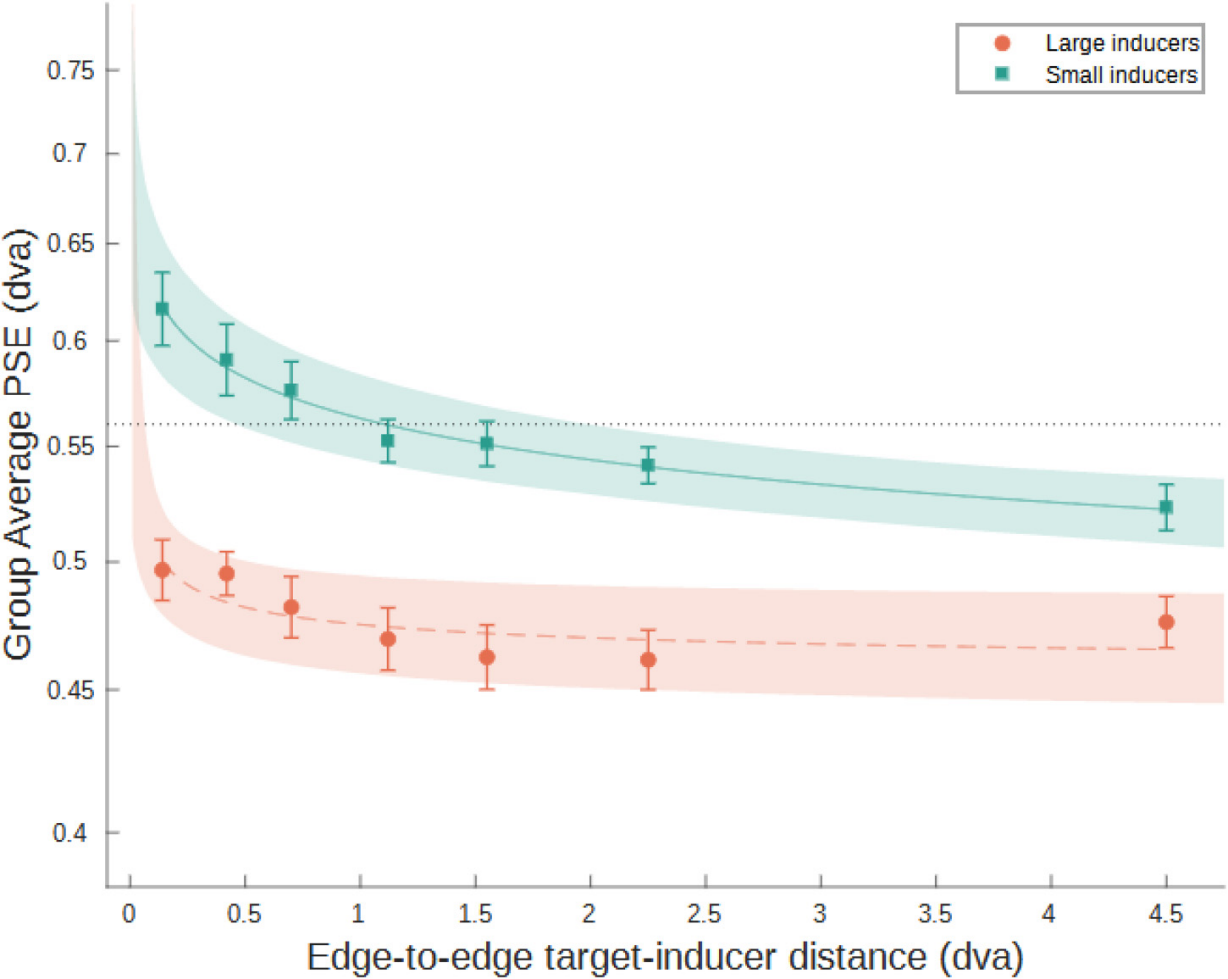
Group mean PSEs across target-inducer retinal distances in Experiment 1. The horizontal dotted black line indicates the size of the reference stimulus, that is, the absence of any illusion. Solid and dashed lines are the fit to the data for the small- and large-inducer conditions, respectively. Shaded regions show the 95% bootstrapped bands for the power functions for each inducer type. Error bars indicate ±1 standard error of the mean across observers. “dva” = degrees of visual angle.

Figure 4 shows the group-level average PSEs for Experiment 1. Prior to analysis, we subtracted the PSE in the control condition from the PSE for both inducer conditions at each distance. These baselined PSEs were used in all subsequent analyses. For both large and small inducers, we fit a power function of the form *ax^b ^+ c*, where *a, b,* and *c* are free parameters and *x* is target-inducer distance. We used a bootstrap technique to calculate the 95% confidence bands for this function by randomly selecting a sample of 12 (with replacement) from the pool of observers and then re-calculating the group means and re-fitting the power function to the new sample. This was repeated for a total of 10,000 times for each inducer type. We calculated goodness-of-fit measures for both small, *R*^2^*^ ^* = .838, and large inducers, *R*^2^*^ ^* = .732, as well as observed model parameters (Supplemental Table 1). A plot containing individual-observer model fits can be viewed in Supplemental Figure 1. In addition to the power function shown here, we also performed the same analysis with a two-term exponential function of the form ae^bx ^+ ce^dx^. We chose this as an alternative model because of the known exponential relationship between eccentricity and cortical magnification (Duncan & Boynton's, 2003). This model performed well with small inducers but we chose a power model here because the exponential model performed poorly with large inducers (see Supplemental Table 2).

Our results support our hypothesis that shorter target-inducer distances lead to an increase in perceived target size (larger, positive PSEs). For targets surrounded by small inducers, there was a clear uptick in PSEs for shorter distances. Moreover, with enough distance the sign of illusion inverted. The pattern for large inducers was more ambiguous. At all target-inducer distances, PSEs were negative, meaning the target was perceived as smaller. Importantly, our results also showed that the basic Ebbinghaus effect occurs with our novel presentation procedure where stimuli are presented near the fovea in separate temporal intervals.

Schwarzkopf and Rees (2013) hypothesized that at a short enough distance to the target, large inducers could make the target appear larger. We tested this by allowing large inducers to overlap and display at a distance much closer to the target compared to Roberts et al.’s (2005) study. Our results did not support this hypothesis, with a modest increase in PSE when large inducers were very close to the target. This may be due to the attractive effect of the nearer contours in large inducers being counteracted by contours on the far side of the inducers (Todorović & Jovanović, 2018), but may also reflect an unanticipated effect of allowing large inducers to overlap at short distances from the target. Specifically, this would also reduce the figural similarity between the inducers and the target, which has been shown to affect the strength of the illusion (Choplin & Medin, 1999; Coren & Enns, 1993; Deni & Brigner, 1997; Jaeger & Guenzel, 2001; Rose & Bressan, 2002).

Generally, our results bear important similarities and differences compared with the results of Roberts et al. (2005). Their study also found a similar pattern when increasing target-inducer distance with large and small inducer conditions. However, they found a more reliable reduction in PSEs at greater target-inducer distances compared to our study. Unlike Roberts et al. we did not vary the numbers of inducers to always form a complete ring around the target, so this discrepancy may reflect lower stimulus energy due to the large distances between them. In both studies, the illusion for small inducers does invert at greater differences, although the crossover point for Roberts et al.'s study (∼3–3.25°) differs considerably to the crossover seen here (∼1.2°). This may be due to changes in illusion strength related to overall stimulus size, a factor shown to reliably effect illusion strength in other studies (Knol et al., 2015; Massaro & Anderson, 1971).

Given that sequential presentation of the elements of the Ebbinghaus illusion can reduce the illusion magnitude (Jaeger & Pollack, 1977), a potential concern stems from our choice to present stimuli at nearby locations. Potentially, an afterimage from the target or inducers from the first interval could affect perception of the second interval; a persistent image of a target may enhance perceived similarity with a second target, and residual images of inducers may introduce an illusory effect on a lone target in the second interval. However, we think these concerns are unlikely for the following reasons. Firstly, observers (including the two authors) did not report seeing afterimages. Secondly, we deliberately offset each interval horizontally (and vertically in Experiment 2, see below), which should reduce the ability for any direct comparisons between stimuli. Thirdly, the temporal order of reference and test stimuli were counterbalanced, meaning any effect of inducers in the first interval would be counteracted by trials where the inducer condition was in the second interval.

Experiment 1 supports the hypothesized relationship between distance in visual space and PSE in the Ebbinghaus illusion. Specifically, we predicted that for a given inducer type (i.e., small, large) as cortical distance between target and inducers decreases, perceived size of the target should increase. We observed this effect, albeit more clearly for small inducers. In Experiment 2 we test the relationship between the Ebbinghaus and cortical distance further by taking advantage of the change in cortical magnification across the visual field.

## Experiment 2

Cortical magnification in visual cortex falls off with eccentricity (Duncan & Boynton, 2003; Smith et al., 2001). Ebbinghaus stimuli at greater eccentricities therefore reduce cortical distance between representations. Chen et al. (2018) found the Ebbinghaus illusion was stronger when observers first viewed a low-spatial frequency prime compared to a high-spatial frequency prime. Sensitivity to low-spatial frequencies increases with eccentricity (Henriksson et al., 2008), and categorization of low-spatial frequency scenes elicits greater activity in brain areas associated with the peripheral visual field compared to high-spatial frequency scenes (Musel et al., 2013). We hypothesize that shorter cortical distances between the target and inducers produce an increase in perceived target size, irrespective of the inducer type. Thus, we should observe generally larger PSEs as stimuli are moved further into the periphery. The effect of eccentricity on the Ebbinghaus illusion has been investigated previously by Eymond et al. (2020). In one experiment, observers in their study compared a foveal test circle with a peripheral or foveal reference circle that was either an isolated control circle or an Ebbinghaus configuration with large inducers. They found the PSE for the Ebbinghaus condition did not differ depending on eccentricity while the control condition appeared smaller in the periphery. While this may initially seem inconsistent with our hypotheses, observers in their study compared a foveal test stimulus with a peripheral target, and the authors note there is a general reduction in perceived size when stimuli are placed into the periphery (Baldwin et al., 2016). Therefore, the lack of an effect of eccentricity on PSE in the Ebbinghaus illusion in their experiment may indicate that the effects of the inducers are counteracting a reduction in perceived size in the periphery. Our experiment differs from these studies in two key ways: Firstly, we test perception of the Ebbinghaus illusion at multiple distances from fixation. This will allow us to observe graded effects of eccentricity. Secondly, targets in reference and test stimuli occurred at the same eccentric location. By doing this, account for stimuli varies in size in absolute terms across the visual field.

### Methods

#### Participants

We recruited 12 observers (10 females, age range 22–53) all with normal or corrected-to-normal vision. Observers provided written and informed consent and procedures were approved by UAHPEC.

#### Experimental Setup

We conducted Experiment 2 on the same experimental setup as Experiment 1 with the addition of an Eyelink 1000 Desktop System eye-tracker (operating at 1,000 Hz; SR Research).

#### Stimuli

The retinal dimensions of target and inducer stimuli were identical to Experiment 1. Unlike Experiment 1, in Experiment 2 retinal target-inducer distances were fixed while we manipulated the location of the Ebbinghaus stimuli along the visual field's horizontal meridian. To avoid crowding, we applied Bouma's law (Bouma, 1970; Pelli & Tillman, 2008), which states that the absence of visual crowding effect can be achieved if the retinal distance between the two visual elements is no less than 50% of the distance between these elements and fixation. Thus, the centers of target stimuli were positioned at a maximum distance of 4.5° from fixation, dictating a suitable target-inducer distance of 2.25°. This distance was used for both large and small-inducer configurations. To avoid influence from attentional capture on each trial, the presentation of the stimuli was preceded by a primer stimulus to alert the observer to the location of the forthcoming stimuli.

#### Procedure

The procedure for Experiment 2 was mostly the same as Experiment 1, except that the retinal distance between target and inducers was kept constant while the distance between the location of the target and foveal vision was manipulated.

Observers sat in a dimly lit room where they positioned their head on a headrest and chinrest apparatus located in front of a computer monitor where they performed a 9-point calibration routine for the eye-tracker. The experimenter verbally instructed observers to maintain fixation on the fixation cross located in the center of the screen, that the eye-tracker was tracking their eyes, and to try to avoid blinking during stimulus presentation time.

Each trial began with presentation of a fixation cross. On a given trial the test and reference target stimuli could either occur at fixation or at an eccentric location close to the horizontal meridian. Eccentric locations could occur either to the left or right hemifield. Exogenous cueing can affect perceived size in the objects in the periphery (Kirsch et al., 2020), so to avoid any extraneous effects of attentional re-orienting, we ensured that attentional allocation was consistent across conditions. We did this with an exogenous cueing stimulus: If on the current trial the target and test targets were to appear at an eccentric location, they were preceded by “×” shaped cue (0.3° × 0.3°) at the location of the forthcoming reference and test stimuli. This cue appeared for 100 ms, followed by a 500 ms interval of only the fixation cross again, followed by the reference and test intervals. The order of test and reference intervals was pseudo-randomly determined on a trial-by-trial basis. Just as in Experiment 1, the reference target stimulus could be either the large- or small-inducer Ebbinghaus configurations or the control stimulus with no inducers, each with a 0.56° diameter target circle. The test stimulus varied according to the same staircase procedure described in Experiment 1. Each interval lasted 100 ms, separated by a 500 ms interval.

Unlike Experiment 1, we offset the location of reference and test target in a vertical (rather than horizontal) orientation to avoid extraneous effects of one stimulus occurring at a more central location than the other. Thus, the center of the target circles in the first and second interval always appeared 0.2° above and below the horizontal meridian, respectively.

We used the eye-tracker to ensure observers were always looking at fixation during presentation of the reference and test stimuli: A trial would be aborted if, during the reference and stimulus intervals, the observer blinked or if their gaze was tracked as deviating more than 1° from fixation. We performed a single-point drift-correction procedure between each 100-trial block.

If the reference and test intervals ran to completion, observers were again shown a fixation cross while they responded by pressing a keyboard button to indicate whether they thought the target in the first (top) or second (bottom) interval was larger or smaller, depending on the instructions of the current block. This response period was untimed and giving a response would immediately initiate the next trial. Consequently, observers were asked to blink and orient their gaze to the fixation cross before giving their response. If the trial was aborted due to blinking or looking away from fixation, the screen would show the fixation cross for 500 ms before initiating the next trial.

There were 12 conditions in total with a 3 × 4 design: three types of inducer conditions (large, small, no inducers) and four eccentricities (0°, 1.69°, 2.8°, and 4.5°). There were two staircases for each of these conditions that operated as in Experiment 1. A given staircase ended after 25 reversals and the whole experiment ended when all staircases reached completion. Each block of trials ended after 100 trials or if all staircases were completed, ending the experiment.

We calculated PSEs for each condition using the same procedure as Experiment 1.

### Results and Discussion

**Figure 5.**
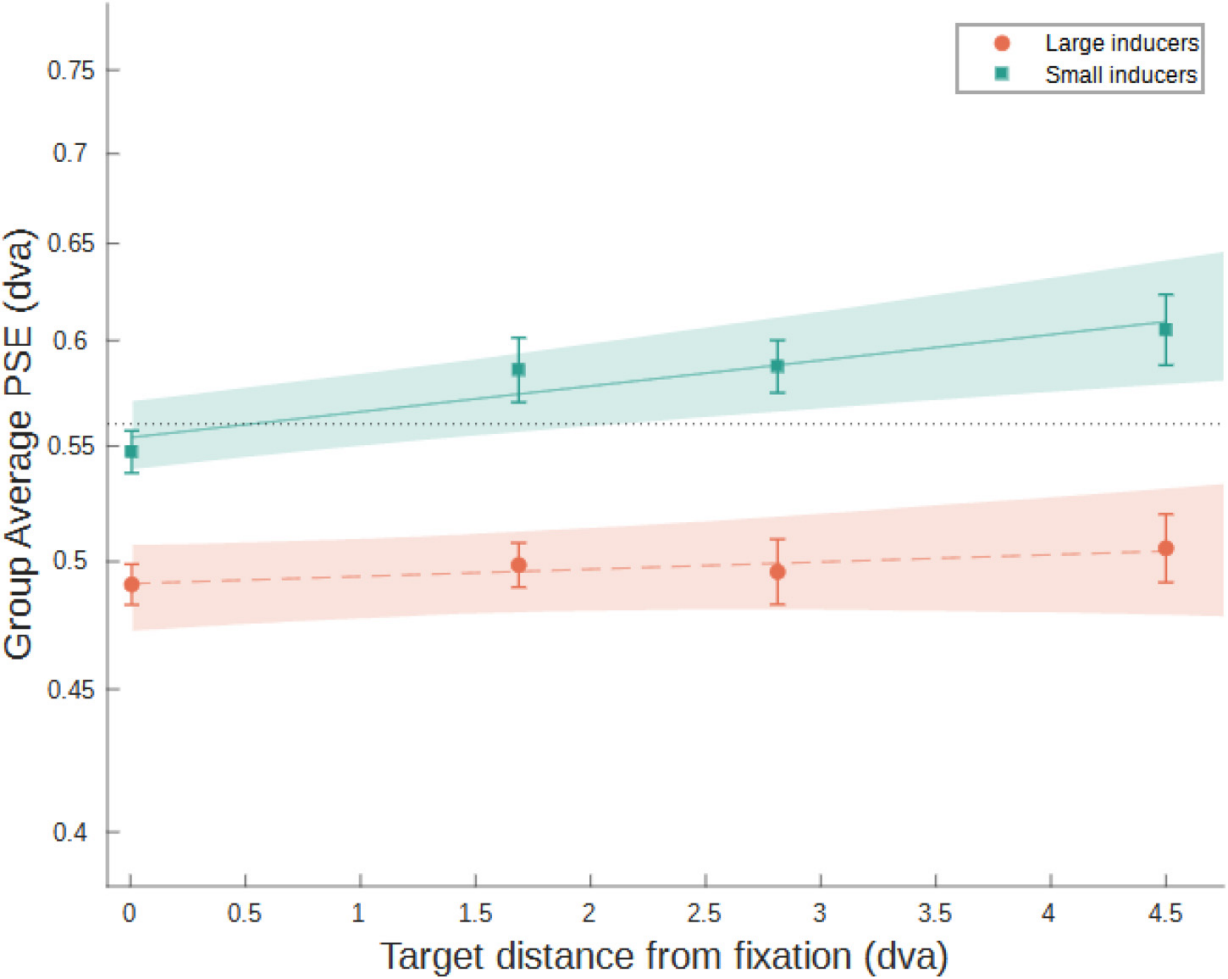
Group mean PSEs (illusion magnitude) across target-fixation retinal distances (eccentricity) in Experiment 2 (units as in Figure 4). The horizontal dotted black line indicates the size of the reference stimulus, that is, the absence of any illusion. Shaded regions show the 95% bootstrapped bands for the linear fit to both types of inducers. Solid and dashed lines show fit to small- and large-inducer conditions, respectively. Error bars indicate ±1 standard error of the mean across observers. “dva” = degrees of visual angle.

As before, for a given eccentricity we subtracted the PSE for the control condition from the PSE for both inducer conditions. These baselined PSEs were used in all subsequent analyses. Figure 5 shows the group-level average PSEs for Experiment 2. Our analysis was to investigate whether PSEs increased or decreased with target eccentricity, and for this purpose we determined a linear function of the form *y* = *a* + *bx* as appropriate, rather than the power function used in Experiment 1. We fit this to the small, *R*^2^*^ ^* = .732, and large, *R*^2^*^ ^* = .839, inducer conditions. Confidence bounds were generated using the same procedure as Experiment 1 and can be found in Supplemental Table 1. Generally, the target appeared larger as target-fixation distance increased, although this effect was not observed to the same extent in large inducers. We see this in the confidence interval for the slope parameter for large inducers, *b* = 0.009 (95% CI [0.025, −0.01]), which overlapped zero. This indicates that there is no clear direction (either positive or negative) of the slope representing the relationship between eccentricity and PSE in the large-inducer condition, and that the slope itself is close to zero. However, the interval for small inducers did not cross zero, *b* = 0.031 (95% CI [0.0445, 0.0166]), indicating a reliable positive relationship between eccentricity and PSE as determined by our bootstrap procedure. We also observed that for some observers the PSE for small inducers switched sign as target-fixation distance increased, in line with our findings while increasing target-inducer distance in Experiment 1.

#### Cortical Distance and PSE

We looked at the effect of cortical distance on the Ebbinghaus illusion. Our approach takes inspiration from Mareschal et al.'s (2010) investigation of the effect of cortical distance on the tilt illusion. The tilt illusion (Gibson, 1937) is an illusion where the perceived tilt of a target line is influenced by the angle of surrounding lines. Mareschal and colleagues estimated cortical distance between target and surround across various retinal distances and concluded that the strength of the tilt illusion increases with cortical proximity. In a similar way, estimates of cortical distance allow us to investigate the relationship between cortical distance and PSE in Experiments 1 and 2. To do this, we chose to estimate linear cortical magnification factor (*M*), which is the millimeters of cortex per degree of visual angle (Daniel & Whitteridge, 1961), using Duncan and Boynton's (2003) formula: *M* = 9.81 × δ^−.083^, where δ denotes eccentricity in degrees of visual angle. By subtracting *M* between two different points (see Mareschal et al., 2010), we can estimate of the cortical distance between inducers and target across inducer conditions and experiments. For stimuli presented at fixation, all inducers in each configuration were equidistant to the target both in terms of visual space and cortical distance. However, when stimuli were presented at parafoveal locations in Experiment 2, the distances between individual inducers and the target were asymmetric; for example, cortical distance from the target is greater for the inducers positioned closer to fixation compared to the more peripherally located inducers. To capture these variations, we calculated an index of cortical distance for each condition based on the average edge-to-edge cortical distance between the nearest edge of all eight inducers and the target.

**Figure 6.**
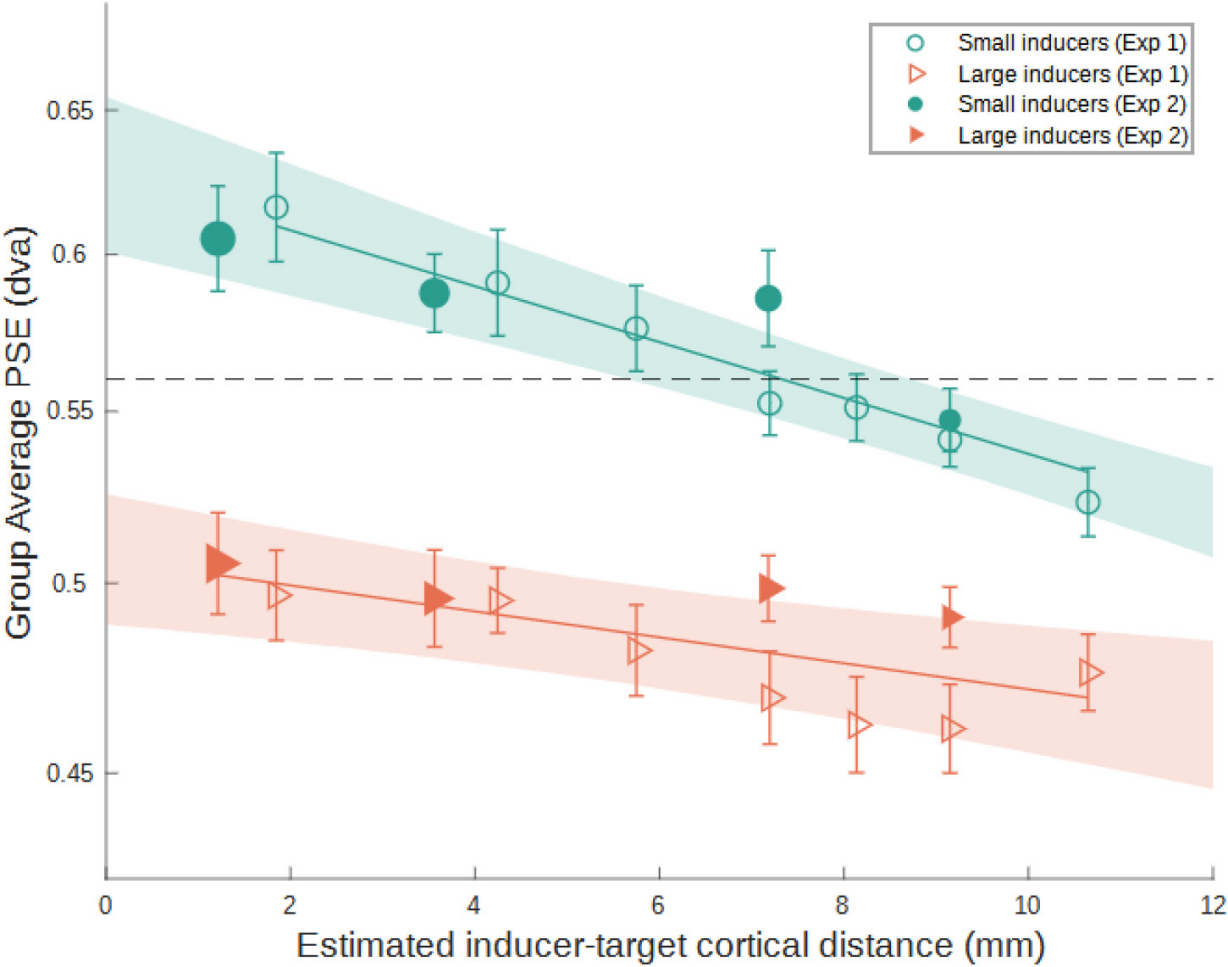
Group mean PSE as a function of estimated cortical distance. The horizontal dashed black line indicates the size of the reference stimulus, that is, the absence of any illusion. Small and large inducers are shown as circles and triangles, and PSEs from Experiment 1 and 2 are denoted by open and filled symbols, respectively. The size of the filled symbols denotes eccentricity in Experiment 2. Error bars indicate standard error (1±) of the mean across observers. Confidence bounds show the 95% upper and lower bounds of the line fit, produced from the bootstrap procedure. “dva” = degrees of visual angle, “mm” = millimeters.

We plot the estimates of cortical distance against PSE in Figure 6. We used a bootstrap method to plot confidence bands by taking 10,000 resamples (with replacement) of observers’ PSEs across both experiments. For the observed and each iteration of the bootstrapped data, we fit a linear function of the form *y* = *a* + *bx*, where *a* is the intercept*,* and *b* is the slope coefficient. This was performed separately for both target-fixation distance and estimated cortical distance. We chose a linear function as a parsimonious way to characterize a simple relationship between two variables. Cortical distance predicted PSE for both small inducers, *R*^2^ = .876, and large inducers, *R*^2^ = .304. For the relationship between cortical distance and PSE, slope coefficients (*b*) for the large and small inducers were −0.01 (95% CI [−0.003, −0.017]) and −0.022 (95% CI [−0.016, −0.028]), respectively. We also ran the same procedure with a Difference-of-Gaussians (DoG) model (see Equation 1), in accordance with our theoretical expectations and to maintain consistency with the analysis in Experiments 3a and 3b (see below). The goodness-of-fit and parameter values (including confidence intervals derived from the bootstrap procedure) can be found in Supplemental Table 3. Upon visual inspection and comparison of goodness-of-fit estimates, we determined that the linear model was a better fit to the data from Experiments 1 and 2. This could be because the data points in the Ebbinghaus experiments fell within the steep portion of this function.

Encouragingly, the two separate methods of manipulating cortical distance between target and inducers had comparable effects on the illusion strength. We observed agreement between PSEs across the two experiments in conditions with similar cortical distance estimates, particularly for the small-inducer condition. Specifically, in Figure 6, markers at a similar position on the *x* axis, irrespective of experiment, have similar PSEs. We observed a negative correlation between cortical distance and Ebbinghaus PSE in both large and small inducers, such that smaller cortical distance corresponded to larger perceived target size. These findings are consistent with the predictions based on previous neuroimaging work showing that smaller cortical extents associate with larger PSEs (Schwarzkopf et al., 2011) and especially perceptually larger stimuli (Schwarzkopf & Rees, 2013). Moreover, the shallower slope seen in the large inducer condition mirrors the results from Experiment 1. This may reflect non-linear interactions between the target and inducers due to antagonistic effects of the near and far contours in large inducers (Todorović & Jovanović, 2018). Mareschal et al.'s (2010) study also described opponent processes, which, in the context of the tilt illusion, were antagonistic “repulsive” and “assimilative” forces. These same mechanisms may account for repulsion and attraction in the Ebbinghaus illusion.

## Experiments 3a and 3b

In the next experiment, we investigate why large and small inducers have contrasting effects on perceived target size. We saw support in Experiments 1 and 2 for the link between PSE and estimated cortical distance, but they also replicated the different perceptual effects of large and small inducers. As these results and others (Roberts et al., 2005; Sherman & Chouinard, 2016; Todorović & Jovanović, 2018) have shown, this disparity is unlikely to be caused by a hypothetical size-contrast effect originating in mid or high-level vision. An alternative account is that these differences are driven by opponent processes which depend on the spatial (or cortical) extent of contours around the target. As others have stated (Rose & Bressan, 2002), contour-based accounts offer an incomplete account of the Ebbinghaus illusion, but it may be necessary. Proponents of accounts such as BCIT hold that this difference can be explained in terms of low-level contour interactions (Jaeger, 1978; Jaeger & Grasso, 1993; Sherman & Chouinard, 2016; Todorović & Jovanović, 2018; Weintraub, 1979; Weintraub et al., 1969; Weintraub & Schneck, 1986). The “biphasic” element of BCIT (Sherman & Chouinard, 2016) stipulates that contours nearer to the target have an attractive effect, while contours at more distance locations repel the target. Thus, the reason large inducers make the target look smaller is because large inducers have additional contours at farther distances from the target. An alternative account for these differences sees them as driven by higher level-categorization of inducers as whole objects beyond simple size contrast (Knol et al., 2015; Rose & Bressan, 2002).

We test the effect of additional contours with the Delboeuf illusion (Delboeuf, 1865; Evans, 1995), an illusion in which perceived target size is affected by the proximity of a ring surrounding the target. The Delboeuf illusion is suitable for this purpose because it likely shares a common mechanism with the Ebbinghaus illusion. Supporting this, both Pressey (1977) and Sherman and Chouinard (2016) found that the two illusions share around a quarter of their variability. In another study, Roberts et al. (2005) found that a complete ring comprised of small Ebbinghaus inducers had the same illusory effect as a Delboeuf ring at a range of distances from the target. Accordingly, if negative PSEs associated with large inducers in the Ebbinghaus illusion are due to near and far contour placement, and if the Delboeuf and Ebbinghaus share a common mechanism, the simple addition of another ring in the Delboeuf illusion (Figure 3) should resemble the effect of large inducers in the Ebbinghaus illusion, such that we observe a downward shift in PSE. We test this hypothesis in Experiment 3a (henceforth “3a”).

In Experiment 3a we observed that PSE did not trend towards zero with greater target-ring distances (see “Results and discussion” section). The interaction between the surround and the target in the Ebbinghaus illusion has been conceptualized as sombrero-shaped center-surround of contextual interactions (Schwarzkopf, 2015), and in such a model we would expect the contextual effects to diminish to zero as it approaches the “brim” of the hat (i.e., the boundaries of any suppressive effect). To this end, in Experiment 3b (henceforth “3b”) we increased the ring-target distance further to observe if its effect on the target attenuates at even farther target-ring distances.

### Methods

#### Participants

We recruited 12 volunteers (seven females, age range 22–49) for 3a and 14 volunteers (9 females, 21–52) for 3b, all with normal or corrected-to-normal vision. Observers provided written and informed consent, and procedures were approved by UAHPEC.

#### Experimental Setup

The experimental setup for 3a was identical to Experiment 1. In order to cover an area of the visual field ∼40° in diameter, we reduced the viewing distance 3b from 82 to 42 cm.

#### Stimuli

In both experiments, the reference-interval stimuli consisted of a central circle (0.56° in diameter) with either a single- or double-ring configuration in 3a (see Figure 3) and a single-ring only condition in 3b. Rings in both experiments had a thickness of ∼0.04°.

For 3a, on a given trial the inner-ring of the double-ring condition could be one of several distances from the edge of the central circle: 0.14°, 0.44°, .7°, 1.13°, 1.55°, 2.25°, and 4.5°. The distances were the same for the single-ring configuration, with the addition of a 5.34° condition (i.e., the distance of the outer ring in the double-ring condition at 4.5°). The distance between the borders of the inner and outer rings was always 0.84°. We chose this distance to match the diameter of the large inducers from Experiment 1, and in doing so emulate the antagonistic effects of near and far contours in those stimuli. Experiment 3b featured the single ring conditions at the following distances: 0.14°, 11°, 15°, and 20°.

#### Procedure

The procedures for both experiments were the same as Experiment 1 (see Figure 2). Observers typically completed the experiment in 45 min for Experiment 3a, and 20 min for 3b.

### Results and Discussion

**Figure 7.**
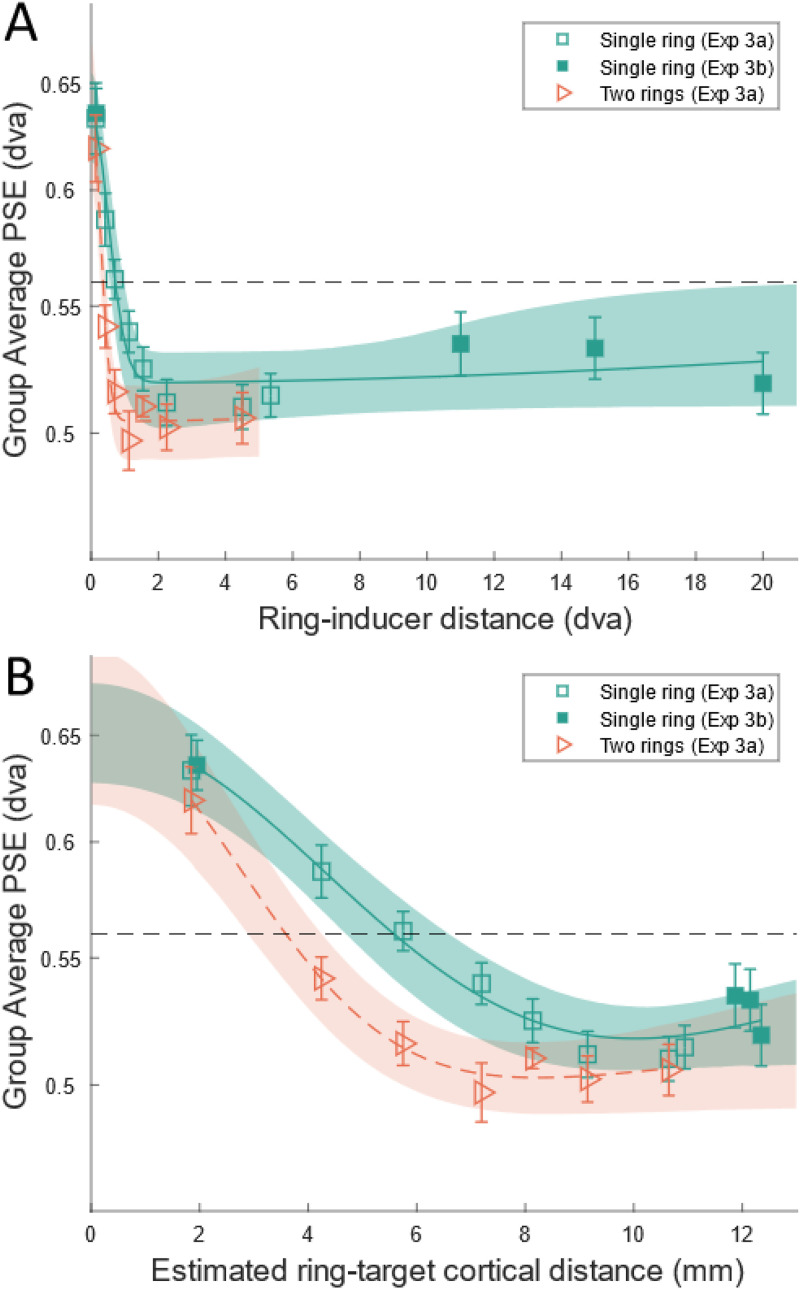
Group mean PSEs across target-ring distances in Experiments 3a and 3b as a function of retinal distance (A) and estimated cortical distance (B). The horizontal dashed black line indicates the size of the reference stimulus, that is, the absence of any illusion. Solid and dashed colored lines show the Difference-of-Gaussians (DoG) function fitted to the data for the two-ring and single-ring conditions, respectively. Shaded regions show the 95% bootstrapped bands for DoG for each inducer type. Error bars indicate ±1 standard error of the mean across observers. “dva” = degrees of visual angle, “mm” = millimeters.

Prior to analysis, we again subtracted the PSE from the control condition from all other conditions and used those baselined PSEs for all subsequent analyses. Figure *7* shows the group-level average PSEs for 3a and 3b as a function of target-ring distance in degrees of visual angle and estimated cortical distance. We also plotted data from Experiments 3a and 3b separately, as PSE as a function of retinal distance and using the same power function used in Experiment 1 (see Supplemental Figure 5 for plots, and goodness-of-fit and parameter estimates in Supplemental Table 1).

In 3a we used the Delboeuf illusion to determine if the difference between large and small inducers in the Ebbinghaus illusion are explainable as an interplay of near and far contours (Sherman & Chouinard, 2016; Todorović & Jovanović, 2018). On this basis our results support our hypothesis; compared to a single ring, the two-ring configuration had the effect of numerically reducing the PSE (Figure 7). Looking at Figure 7b, this downward shift is most pronounced for cortical distances between 2 and 8 mm. Biphasic-contour interaction theory explains this as a result of antagonistic effects of contours, with farther contours working to repulse the percept of the target. At the largest cortical distances, the two rings probably fall within the same large peripheral receptive fields. Thus, the two-ring condition may effectively be a single-ring condition at these distances, only with somewhat increased stimulus energy. Our results also agree with earlier research, albeit with updated psychophysical methods. For example, with a target-inducer distance comparable to our own, Weintraub and Schneck (1986) observed that the target appeared larger when only the inner-arc of large inducers were visible, but that PSE decreased and eventually changed sign as the outer fragments of those inducers were filled in with successively more dots. This resembles the addition of the outside contour in 3a, which we observed as shifting the PSE for most target-ring distances.

Similarities aside, the effects of the two ring conditions in this experiment and those of small and large inducers in Experiment 1 (Figure 4) are not an exact match. This may be explained in terms of a contour-based account, as these two illusions differ in terms of variations in stimulus energy (i.e., the amount of contour). Unlike Roberts et al. (2005), we did not modify the number of inducers to maintain an uninterrupted surround of inducers in our experiments, meaning that at all distances the Delbouef presented a more complete surround than in Experiment 1. By contrast, Roberts and colleagues found that an uninterrupted surround of small inducers affected perceived target size almost identically to a Delboeuf ring at various distances from the target. The two conditions in 3a had the same effect at very close distances (0.14°), yet there was a total separation of PSEs between the large- and small-inducer conditions at that same distance in Experiment 1. This, too, may be attributable to a difference in stimulus energy because of intermediary contours between the nearest and farthest edges in large inducers, which are absent in the Delboeuf illusion. Alternatively, the difference we observe between these two illusions may indicate a contribution of a second mechanism, possibly located higher in the visual stream. We discuss these possibilities further in the General discussion.

#### DoG Function

We modelled the relationship between PSE and retinal and cortical distance, respectively, using a DoG function. DoG has been used to model inhibitory signals in extra-classical receptive fields (Cavanaugh et al., 2002), and it can account for the antagonistic center-surround proposed by Schwarzkopf (2015) and in BCIT (Sherman & Chouinard, 2016; Todorović & Jovanović, 2018; Weintraub & Schneck, 1986). To model a center-surround, we calculated the difference of two Gaussian functions with peaks at zero distance. This took for form of Equation 1, where values *σ_a_*, *σ_b_*, *a*, and *b*, are left as free parameters. The DoG function is also used here to investigate whether the relationship between cortical distance and PSE resembles the hypothesized profile of an antagonistic center-surround mechanism. We fit this function using a least-squares procedure and generated 95% confidence bounds using a bootstrap technique with 10,000 repetitions. A single function was fit to the combined PSEs from the single-ring condition in Experiments 3a and 3b, and another on the two-ring condition from 3a.

For PSE as a function of retinal distance we fit DoG functions for small ring, *R*^2 ^= .955, and large inducer conditions, *R*^2 ^= .99. For PSE as a function of cortical distance we did the same for the small ring, *R*^2 ^= .974, and large ring conditions, *R*^2 ^= .997.

(1) $$f(x)=ae^{\frac{-x^{2}}{2\sigma_{a}^{2}}}-be^{\frac{-x^{2}}{2\sigma_{b}^{2}}}$$

Equation 1: DoG function

We observe something closely resembling the sagittal cross-section of a sombrero, as described by Schwarzkopf (2015), with an excitatory center and inhibitory surround (Cavanaugh et al., 2002). Due to a theoretical interest relating to the cortical point image (see General discussion), we also calculated zero-crossing for the two functions in Figure 7B (PSE as a function of cortical distance), with 95% confidence bounds generated from the bootstrapping procedure. That crossing was 5.57 mm (95% CI [6.53 4.25]) for the small ring and 3.65 mm (95% CI [4.76 2.98]) for large ring condition.

An implication of a putative center-surround zone of interaction (Schwarzkopf, 2015) is a non-monotonic relationship between cortical distance and PSE. Theoretically, the effect should reduce to zero at sufficiently large cortical distances as the interaction between contours drops off. Mareschal et al. (2010) found such a “sombrero” pattern when varying cortical distance in the tilt illusion. In our study, even the farthest distances in 3b that did not return to zero. In aggregate, PSEs for distances between 11° and 20° (the three filled green squares on the right side of Figure 7) averaged slightly closer to zero in log units, that is, no illusion (*M *= −0.083, *SE *= 0.029), than the farthest single-ring condition in 3a (5.34°) (*M *= −0.123, *SE *= 0.024), although the difference between these was not significant, *t*(24) = 1.045, *p* = .307. Hence, the PSEs across 3a and 3b trend towards zero, but the cortical distance necessary to see this is evidently beyond the dimensions we measured. We note that the results from 3b are not inconsistent with a contour-based account. Specifically, because of cortical magnification (Duncan & Boynton, 2003), the large distances used in 3b translate to only minor differences in cortical distance compared to 3a (see Figure 7).

## General Discussion

Numerous studies and many theories have been put forward to explain the Ebbinghaus and Delboeuf illusions (as many as 10; for a review, see Robinson, 1998). Despite that, little is known about what lies behind the illusion, and where in the brain that mechanism occurs. The present research adds to a growing body of research pointing to striate cortex as a promising location for this substrate. Such work shows that V1 encodes perceived size, like in the cases of the hallway illusion (Murray et al., 2006), and retinal afterimages projected onto near and far surfaces (Sperandio et al., 2012). Additionally, there is partial interocular transfer of the Ebbinghaus illusion (Song et al., 2011), suggesting a cortical process and again implicating V1, as this region is partially monocular, although of course this could also involve monocular and binocular neurons across multiple stages of the visual hierarchy (Dougherty et al., 2019). Finally, there is a correlation between PSE and between-subject variability in cortical magnification (Schwarzkopf et al., 2011; Schwarzkopf & Rees, 2013). Based on these findings, Schwarzkopf and Rees (2013) and later Schwarzkopf (2015), raised the possibility that interaction between representations on the topography of V1 may be the substrate for the Ebbinghaus illusion, and that these interactions depend in part on the cortical distance between those interactions. This proposal offers a plausible neural basis for contour-based accounts of the Ebbinghaus illusions, in which the low-level interactions between contours underlies the effect. To query this theory further, in the first two experiments we tested the effect of cortical magnification on the Ebbinghaus illusion.

Experiment 1 replicated previous work showing that shorter retinal distances between targets and Ebbinghaus inducers increases PSE (Knol et al., 2015; Roberts et al., 2005). Following this, Experiment 2 showed that for small inducers PSE increases when the target is positioned more peripherally. The prevailing trend in both experiments is consistent with the predictions that (1) in a general sense, the Ebbinghaus illusion depends in part on cortical magnification, and the specific prediction that (2) PSE correlates negatively with cortical distance. We were able to show converging evidence to support this claim by manipulating cortical distances in two ways: firstly, by adjusting the retinal distance between target and inducer (Experiment 1), and secondly, by taking advantage of a reduction in cortical magnification across the visual field and displaying targets in the periphery (Experiment 2). This culminated in the combined results of both experiments and comparing PSE against the estimated cortical distance between targets and inducers (Figure 6). In Experiment 3a, we used the Delboeuf illusion to show that, compared to a single-ring condition, a two-ring condition produced a perceptual shrinkage of the target (PSEs shifted down), lending support to a biphasic-contour account where more distal contours cause a decrease in the perceived size of the target.

The attractive or repulsive effect of Ebbinghaus-style inducers may depend on a cortical distance equivalent to the cortical point image. The point image is the cortical representation of a single point stimulus expressed in millimeters (Mcllwain, 1986), calculated as the product of the cortical magnification factor and receptive field size. Harvey and Dumoulin (2011) used pRF mapping to show that the cortical point image is near constant in V1, with only small decreases with eccentricity (similar findings are reported in non-human primates (Palmer et al., 2012). That is, in V1, there is a constancy in the ratio between receptive field size and cortical magnification. Looking at the Ebbinghaus illusion, Schwarzkopf and Rees (2013) found that perceived enlargement of the target in a small-inducer Ebbinghaus configuration occurred in observers with a relatively small V1. Using similar calculations to those used here (Duncan & Boynton, 2003), they estimated the cortical distance between target and inducer in their study (small inducer condition) to be ∼3.3 mm, falling within the 3–4 mm range of given for point images in V1 (Harvey & Dumoulin, 2011). Schwarzkopf and Rees surmised that the stability of the point image across the cortex indicates “constancy in spatial extent of cortical responses” (p. 11), and that the critical cortical distance between target and inducers in order for a perceived enlargement (i.e., attraction between contours) to occur might be equivalent to the cortical point image. Of interest here is whether a shift between attraction and repulsion occurs at a similar cortical distance in the present experiments. We estimated cortical distance at which a sign change (i.e., from perceived smaller to perceived larger) occurs as ∼7.2 mm for the small inducers in Ebbinghaus illusion (Experiment 1 & Experiment 2). These differ considerably to point image size in V1, which may reflect influences of surround completeness. Moreover, other studies have shown (Knol et al., 2015), target size influences PSE, so these differences may also reflect a difference in overall target size, which was 1.03° in Schwarzkopf and Rees's study, compared to 0.56° in our study. Additionally, the arrangement of small inducers in their study were smaller (small inducer diameter was 26% of target diameter) compared to those used in the current study (35% of target diameter) and formed a more complete ring around the target compared to our study. The relative size of each inducer and completeness of the ring formed are known influences on PSE (Roberts et al., 2005). Supporting this is that when we used the Delboeuf illusion, which consists of an uninterrupted ring, the sign change occurred between ∼5.5 and ∼3.7 mm for the single and double ring conditions, respectively (Experiment 3), which are closer to the estimates of Schwarzkopf and Rees. Thus, if the cortical point image is relevant to the magnitude with these illusions it likely interacts with overall stimulus energy.

Despite our evidence for a link between cortical distance and the Ebbinghaus and Delboeuf illusions, conversion of retinal distances into cortical distance does not completely account for strength of illusions in other studies. As mentioned above, direct comparisons between the present experiments and those of Schwarzkopf and Rees (2013) and other comparable works (Knol et al., 2015; Roberts et al., 2005) are complicated by differences in stimulus dimensions and placement. For instance, Schwarzkopf and Rees (2013), Roberts et al. (2005), and Knol et al. (2015) presented reference and target stimuli at a distance (distances from fixation to target center: 4.65°, ∼15.2°, and 13°, respectively) at *either side* of fixation, whereas we (with a few exceptions) presented stimuli at the same foveal locations at separate intervals. Compared to the experiments here, PSEs are not affected by cortical distance in the same way in those experiments. For instance, one of the conditions in Knol et al.'s (2015) study showed a repulsive effect of inducers (i.e., the target appeared larger) with a 0.48° target, a 1.9° target-inducer distance (measured as distance between target and inducer centers), and inducer radius of 0.09°. Considering the location of the target center in that condition (15.2° horizontal displacement from fixation) and using Duncan and Boynton's (2003) formula, the estimated cortical distance between targets and the nearest edge of the farthest inducers along the horizontal meridian averages to 0.198 mm. This is well within the range where attraction occurred in our experiments, yet in their experiment this distance coincided with a shrinkage of perceived target size.

There are several factors that may account for these differences. Firstly, the stimuli in Roberts et al. (2005), Schwarzkopf and Rees (2013), and Knol et al. (2015) may have been affected by crowding effects; all three studies chose horizontal eccentricities and target-inducer spacing was generally shorter in those experiments than what Bouma's law (Bouma, 1970) would dictate as necessary to avoid crowding. Indeed, there is evidence that size perception (as opposed to only recognition), is affected by crowding (van den Berg et al., 2007). Crowding thus likely interferes with discriminating peripheral target sizes presented in close proximity to the inducers. This would render measurements of PSEs more variable and potentially obscures other effects. Knol et al. and Schwarzkopf and Rees both failed to find substantial repulsive effects on perceived target size, yet Roberts et al., who used large horizontal displacements of their targets, reported an attractive effect at short target-inducer distances with small inducers, and effect resembling our findings from Experiment 1 when stimuli were presented at fixation. It is also a distinct possibility that attractive effects in the illusion and crowding share the same underlying neural mechanism. When cortical distance is sufficiently large, this would result in a perceptual overestimation of the target, but when cortical distance is too short it completely disrupts size discrimination. Finally, are potential differences originating in the task: In addition to greater horizontal displacement, Roberts et al., Schwarzkopf and Rees, and Knol et al. also presented stimuli simultaneously, while we presented stimuli in separate temporal intervals.

We stress that a low-level contour interaction underlying two size illusions of the nature we describe here may fall short of providing a complete account of these illusions. Two decades ago, Rose and Bressan (2002) observed that research with inducers the same size or larger than the target does not have effects on perceived size consistent with a static zone of repulsion and attraction (Girgus et al., 1972; Jaeger & Grasso, 1993; Massaro & Anderson, 1971; Weintraub, 1979). This is reflected in the current study, with the large inducer condition being less responsive than small inducers to manipulations of retinal size and eccentricity in Experiments 1 and 2, respectively. A constant gradient of interaction between contours would not explain this discrepancy between conditions, even when considering potential antagonistic effects between relatively near and far contours. This is even more apparent when comparing Experiment 1 (Ebbinghaus illusion) with Experiment 3 (Delboeuf illusion). We have suggested that these inconsistencies may be attributable to less consistent spacing (and thus differences in stimulus energy) of Ebbinghaus inducers versus the continuous ring in the Delboeuf illusion, but mid- and high-level cognitive factors may also fill this explanatory gap.

Last but not least, several studies suggest that cognitive factors modulate the strength of size illusions, including the Ebbinghaus illusion. For example, Gestalt grouping of a surround reduces PSE in the Ebbinghaus illusion (Rashal et al., 2020), and several studies have observed that size is affected by figural similarity between inducers and targets, even while controlling for the proximity and distribution of contours (Choplin & Medin, 1999; Coren & Enns, 1993; Deni & Brigner, 1997; Jaeger & Guenzel, 2001; Rose & Bressan, 2002). These figural effects have commonly been explained in terms of object-level categorization and attention. Indeed, attention in other contexts has been known to affect perceived size; Kirsch et al. (2020) found that a peripheral target appears small while attending to a central target. Fang et al. (2008) used fMRI to show that spatial distribution of activity in V1 reflected the perceived size of two attended targets embedded in the hallway illusion, and that this activity was significantly diminished when attention was narrowed by a demanding central task. There are also reports that semantic knowledge affects the Ebbinghaus illusion, with objects of a known size biasing their perceived size when surrounded by Ebbinghaus-style inducers (Hughes & Fernandez-Duque, 2010). This relates to findings that ventral temporal cortex (Konkle & Oliva, 2012) show selectivity for real-world size of objects, independent of image transformations. Consistent with known models of predictive processing across various stages of visual processing (Ballard & Jehee, 2012; Rao & Ballard, 1999), these size-encoded representations could drive feedback signals that boost predicted signals in earlier visual areas in V1 consistent with these predictions. Thus, while a mechanism that involves spatially contingent interactions between low-level representations shows promise in explaining some of the known characteristics of the Ebbinghaus and Delboeuf illusions, more work is needed to determine whether contour-interaction alone can explain these illusions or if there is a need for the addition of other (potentially cognitive) mechanisms.

### Conclusion

In addition to showing a relationship between cortical distance and the Ebbinghaus illusion (Schwarzkopf & Rees, 2013), our results broadly support biphasic contour-based accounts of the Ebbinghaus and Delboeuf illusions. Specifically, shorter cortical distances between inducers and target in the Ebbinghaus illusion, whether due to retinal distance (Experiment 1) or cortical magnification (Experiment 2), associate with perceptual enlargement of the target. We did not confirm the prediction that large Ebbinghaus inducers produce perceptual enlargement at short distances, a finding potentially due to repulsive effects of distal contours (on the far side of the inducer relative to the target) counteracting attractive effects of nearer contours. We noted that predictions based on estimated cortical distance did not align with select findings from other studies (Knol et al., 2015; Roberts et al., 2005; Schwarzkopf & Rees, 2013), possibly due to differences in stimulus dimensions, stimulus location, and task design. In the Delboeuf illusion, we showed that the addition of a second, more distant contour reliably decreases perceived target size compared to a single-ring, a finding aligned with an antagonistic surround described in contour-based accounts, such as BCIT (Roberts et al., 2005; Sherman & Chouinard, 2016; Todorović & Jovanović, 2018; Weintraub & Schneck, 1986). Lastly, we found that at large retinal distances (>11°), a single-ring Delboeuf ring still decreased perceived target size, a finding that may reflect low cortical magnification (the relatively minor changes in cortical distance) at points ranging into peripheral vision. Future studies should continue to characterize effects of the surround in these illusions, including interactions between surround elements, potential influences of task design, and contributions of mid- and high-level vision.

## Supplemental Material

sj-pdf-1-pec-10.1177_03010066231175014 - Supplemental material for Effects of cortical distance on the Ebbinghaus and Delboeuf illusions Click here for additional data file. Supplemental material, sj-pdf-1-pec-10.1177_03010066231175014 for Effects of cortical distance on the Ebbinghaus and Delboeuf illusions by Poutasi W. B. Urale and Dietrich Samuel Schwarzkopf in Perception
