# Supplementary material for: Effects of cortical distance on the Ebbinghaus and Delboeuf illusions
Source: Perception. 2023 Jun 19;52(7):459–83. doi: 10.1177/03010066231175014 (PMC10291393; doi:10.1177/03010066231175014)
Supplement: sj-pdf-1-pec-10.1177_03010066231175014 - Supplemental material for Effects of cortical distance on the Ebbinghaus and Delboeuf illusions [file sj-pdf-1-pec-10.1177_03010066231175014.pdf]

Supplementary information

1.1 Supplementary information table of contents

Supplementary Figure 1. Individual-observer fits for participants in Experiment 1.2

Supplementary Figure 2. Individual-observer fits for participants in Experiment 2.3

Supplementary Figure 3. Individual-observer fits for participants in Experiment 3a..4

Supplementary Figure 4. Individual-observer fits for participants in Experiment 3b.5

Supplementary Figure 5. Group mean PSEs across target-inducer distances in Experiments 36

Supplementary Table 1. Model parameters for all experiments.7

Supplementary Table 2. Model parameters for alternative exponential models for experiments 1, 3a, and 3b.8

Supplementary Table 3. Model parameters for cortical distance/retinal distance*PSE plots (Ebbinghaus)9

Supplementary Table 4.10

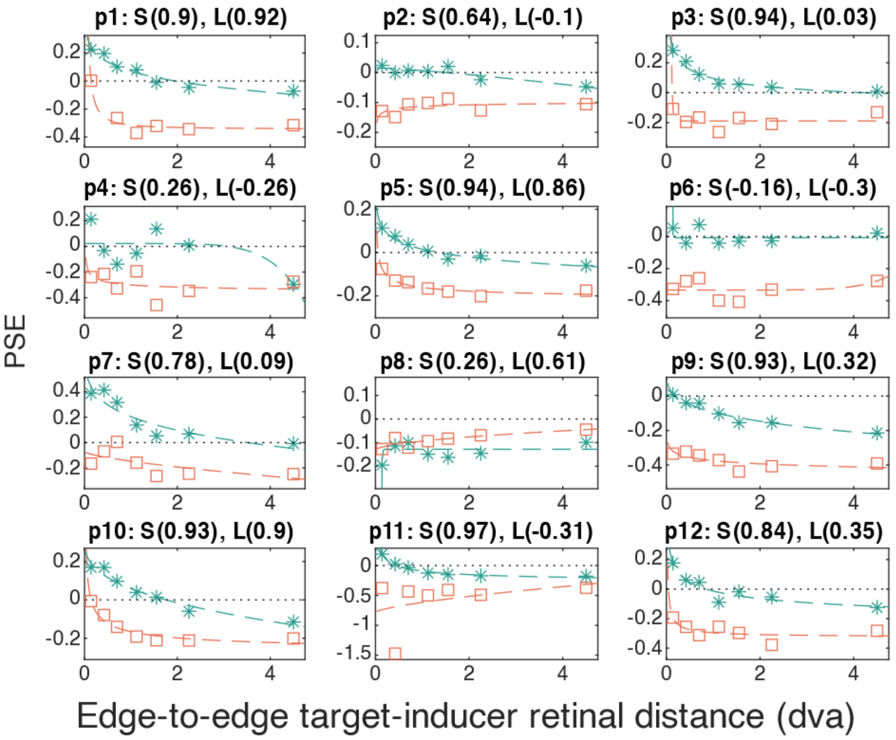

Supplementary Figure 1. Individual-observer fits for participants in Experiment 1. Asterix and square symbols indicate PSEs for the small- and large-inducer conditions, respectively. Titles of each subplot indicate the observer number, followed by the adjusted R^2 values for small (S) and large (L) conditions. “dva” = degrees of visual angle.

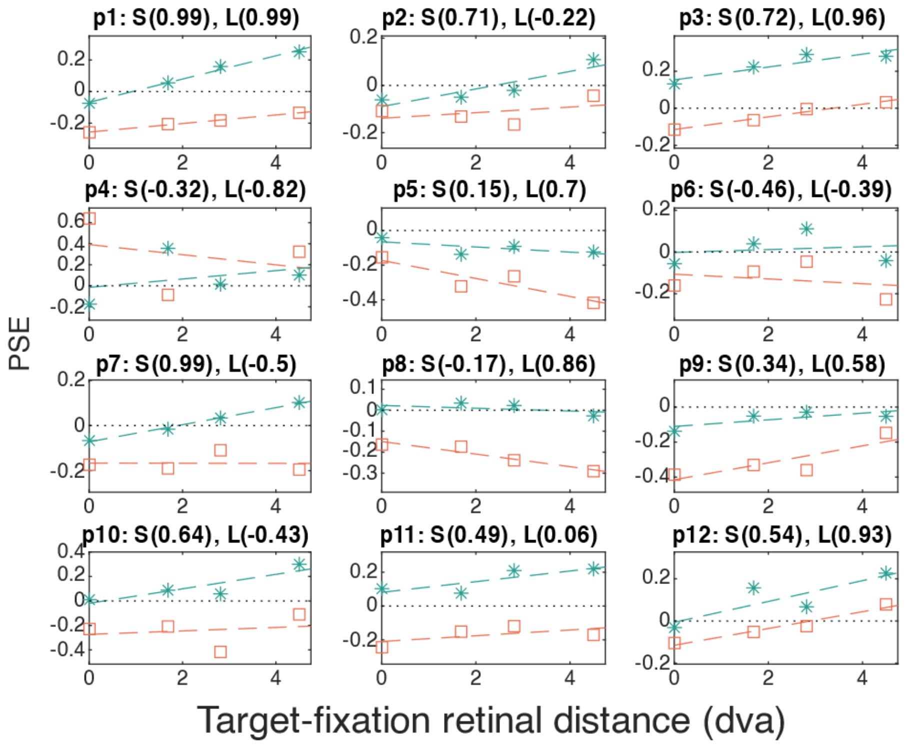

Supplementary Figure 2. Individual-observer fits for participants in Experiment 2. Asterix and square symbols indicate PSEs for the small- and large-inducer conditions, respectively. Titles of each subplot indicate the observer number, followed by the adjusted R^2 values for small (S) and large (L) conditions. “dva” = degrees of visual angle.

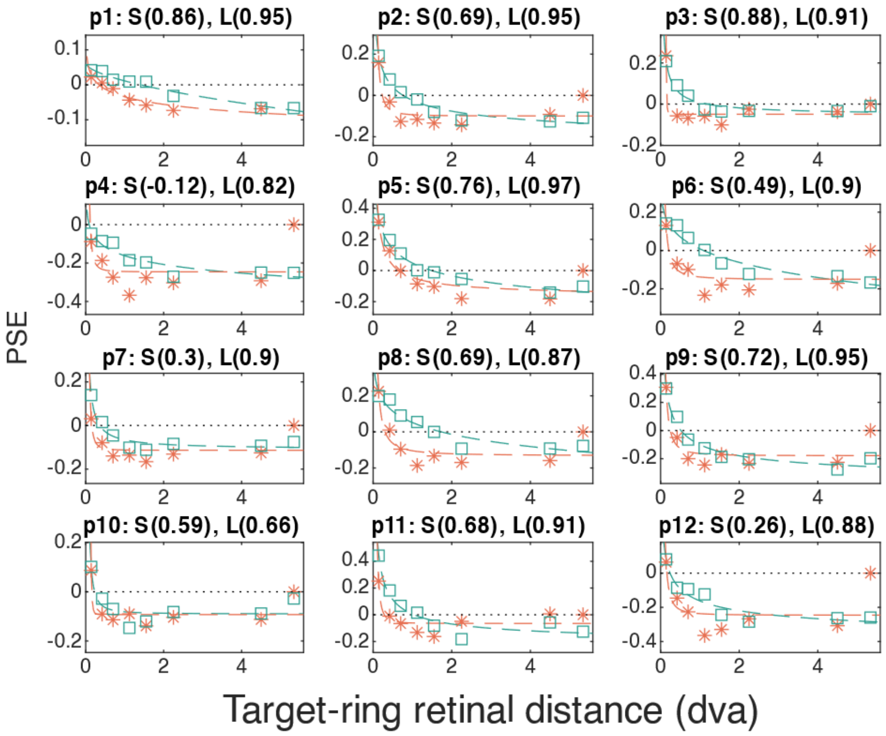

Supplementary Figure 3. Individual-observer fits for participants in Experiment 3a. Asterix and square symbols indicate PSEs for the small- and large-inducer conditions, respectively. Titles of each subplot indicate the observer number, followed by the adjusted R^2 values for small (S) and large (L) conditions. "dva" = degrees of visual angle.

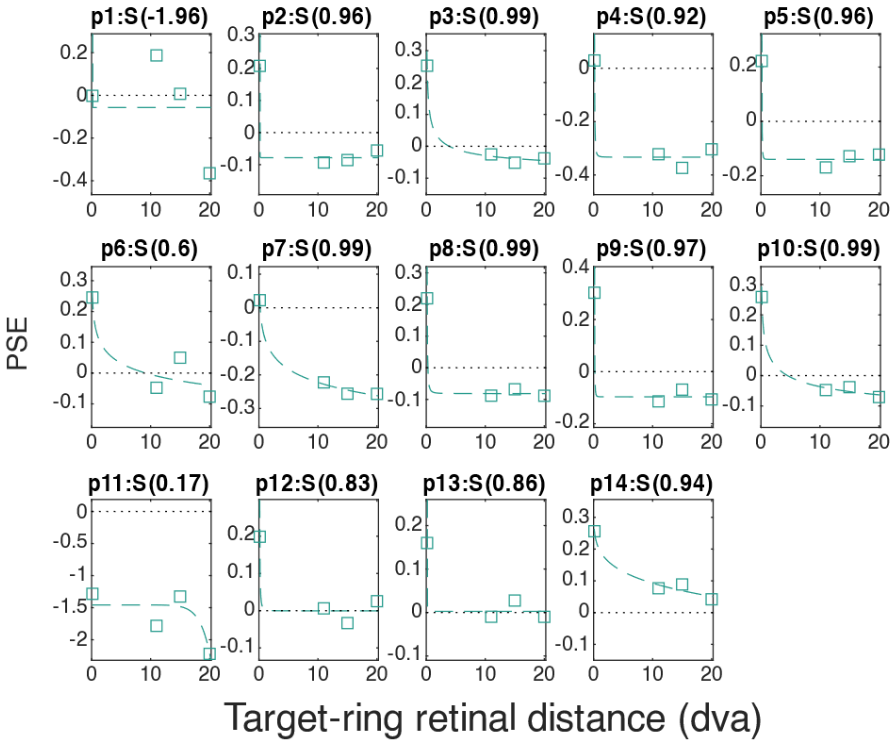

Supplementary Figure 4. Individual-observer fits for participants in Experiment 3b. Asterix and square symbols indicate PSEs for the small- and large-inducer conditions, respectively. Titles of each subplot indicate the observer number, followed by the adjusted R^2 values for small (S) and large (L) conditions. “dva” = degrees of visual angle.

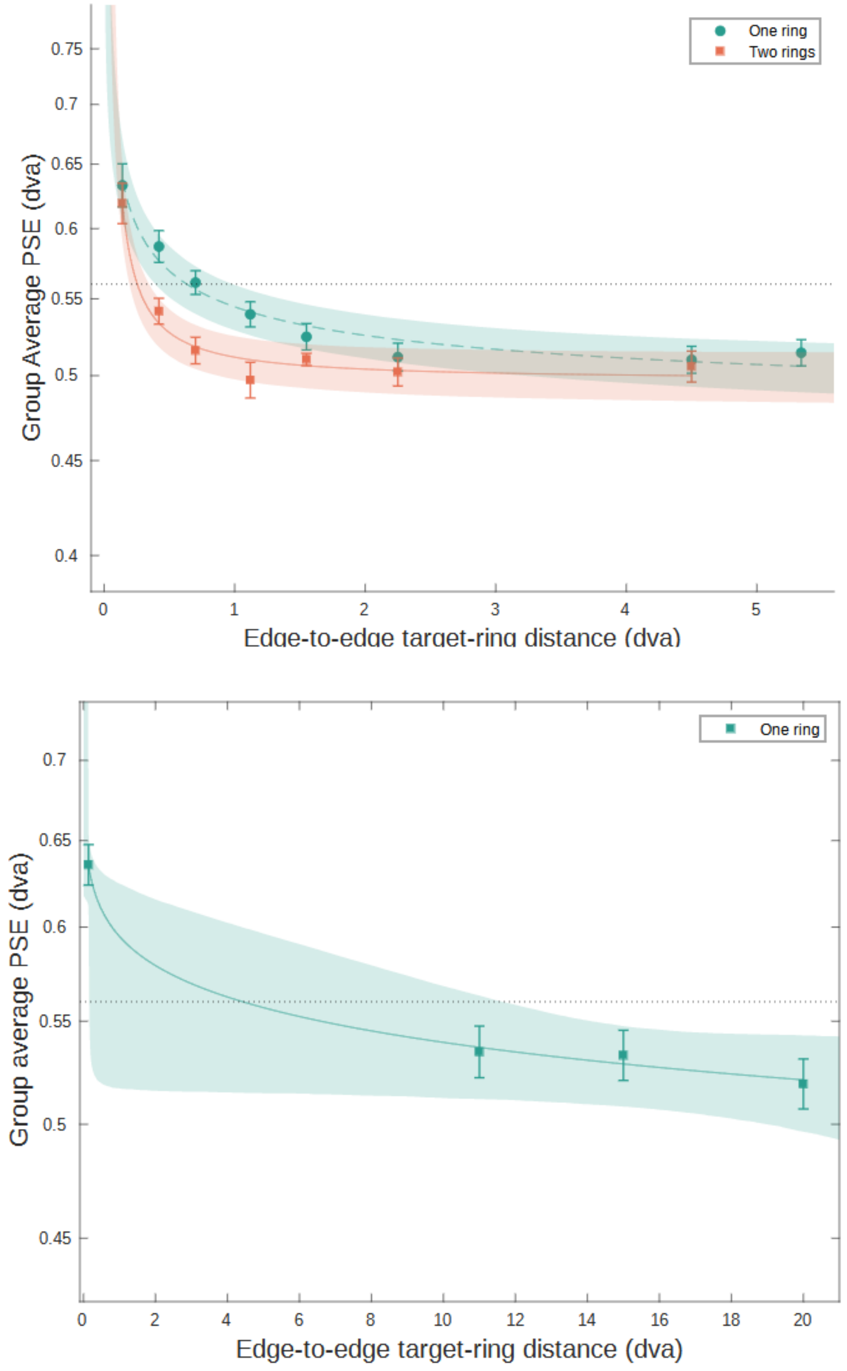

Supplementary Figure 5. Group mean PSEs across target-inducer distances in Experiments 3a (top panel) and 3b (bottom panel). Solid and dashed lines are the fit to the data for the small- and large-inducer conditions, respectively. Shaded regions show the 95% bootstrapped bands for the DoG functions for each inducer type. Error bars indicate ±1 standard error of the mean. Goodness-of-fit measures, model parameters, and confidence intervals for both experiments can be found in **Supplementary Table *1***. “dva” = degrees of visual angle.

Supplementary Table 1. Model parameters (observed and bootstrapped 95% CI) for all experiments.

Experiment 1: Ebbinghaus-distance, $y = ax^b + c$

	Large inducers			*Small inducers*		
	observed	*97.50%*	*2.50%*	*observed*	*97.50%*	*2.50%*
a	0.06759	0.6494	-0.3435	-2.22	9.3063	10.3302
b	-0.3819	0.0637	-0.8584	0.03168	0.2256	-0.1838
c	-0.3056	0.1374	-0.8838	2.227	10.3234	-9.2746
Adj R^2	0.73176			0.83846		

Experiment 2: Ebbinghaus-eccentricity, $y = a + bx$

	Large inducers			*Small inducers*		
	observed	*97.50%*	*2.50%*	*observed*	*97.50%*	*2.50%*
a	0.008598	0.0252	-0.0098	0.03062	0.0445	0.0166
b	-0.1898	-0.1434	-0.2463	-0.01586	0.0273	-0.0538
Adj R^2	0.839			0.732		

Experiment 3a: Delboeuf, $y = ax^b + c$

	Single-ring			*Two rings*		
	observed	*97.50%*	*2.50%*	*observed*	*97.50%*	*2.50%*
a	0.253	0.6642	0.1199	0.04189	0.0901	0.0168
b	-0.3255	-0.1255	-0.5386	-1.033	-0.7194	-1.4331
c	-0.2934	-0.1676	-0.6923	-0.1723	-0.1249	-0.2285
Adj R^2	0.95409			0.95452		

Experiment 3b: Delboeuf plus, $y = ax^b + c$

	Single-ring		
	observed	*97.50%*	*2.50%*
a	-0.4925	2.4054	-3.0581
b	0.1096	1.0306	-8.8303
c	0.5796	3.1345	-2.3412
Adj R^2	0.989		

Supplementary Table 2. Model parameters (observed and bootstrapped 95% CI) for alternative exponential models for experiments 1, 3a, and 3b.

Experiment 1: Ebbinghaus-distance, $ae^{bx} + ce^{dx}$

	Large inducers			*Small inducers*		
	observed	*97.5%*	*2.5%*	*observed*	*97.5%*	*2.5%*
a	-0.032	446.464	-462.737	-0.037	49.383	-44.438
b	0.248	0.643	-0.37	0.531	1.081	-2.223
c	0.215	462.615	-446.617	0	45.021	-49.215
d	-1.568	0.622	-1.255	0.531	0.752	-4.498
Adj R^2	-31.6272			0.98532		

Experiment 3a: Delboeuf, $ae^{bx} + ce^{dx}$

	Single-ring			*Two rings*		
	observed	*97.5%*	*2.5%*	*observed*	*97.5%*	*2.5%*
a	-0.164	-0.117	-0.204	0.407	0.491	-0.317
b	-0.026	0.027	-0.127	-1.258	0.09	-1.448
c	0.514	0.647	0.36	-0.17	0.526	-0.227
d	-3.636	-2.776	-4.595	-0.055	0.09	-1.756
Adj R^2	0.99661			0.97552		

Experiment 3b: Delboeuf plus, $ae^{bx} + ce^{dx}$

	Single-ring		
	observed	*97.5%*	*2.5%*
a	-0.031	0	-0.221
b	0.062	0.292	-0.049
c	0.251	0.574	0.155
d	-1.138	0.574	0.155
Adj R^2	NA		

Supplementary Table 3. Model parameters (observed and bootstrapped 95% CI) for cortical distance*PSE plots (Ebbinghaus). Parameters are given for both a linear fit and Difference-of-Gaussians function. Difference-of-Gaussians parameters are provided in the main text (Equation 1).

Ebbinghaus, Difference of Gaussians, cortical distance*PSE

	Large inducers			*Small inducers*		
	observed	*97.5%*	*2.5%*	*observed*	*97.5%*	*2.5%*
a	1.209351	64.37933	-2.5E+07	0.73446	217.1237	0.228633
σ_a	1.360072	2.055306	0.192978	0.615052	217.0002	0.111026
b	7.537514	11.22373	-9.72081	12.84704	239.8847	5.614097
σ_b	9.086185	3.43E+08	7.843755	9523196	33692593	12.38043
Adj R^2	0.060783			0.895109		

Ebbinghaus, $y = a + bx$, **cortical distance*PSE**

	Large inducers			*Small inducers*		
	observed	*97.5%*	*2.5%*	*observed*	*97.5%*	*2.5%*
a	-0.14446	-0.0921	-0.1964	0.163604	0.2257	0.1015
b	-0.0104	-0.0039	-0.0168	-0.02237	-0.0163	-0.0283
Adj R^2	0.429			0.876		

Supplementary Table 4. Model parameters (observed and bootstrapped 95% CI) for cortical distance/retinal distance*PSE plots (Delboeuf). Difference of Gaussians parameters are provided in the main text (Equation 1).

Delboeuf, Difference of Gaussians, cortical distance*PSE

	Single-ring			*Two rings*		
	observed	*97.5%*	*2.5%*	*observed*	*97.5%*	*2.5%*
a	0.410104	0.556818	0.312758	4.145934	7.986724	0.324038
σ_a	0.187176	0.295688	0.137961	3.929253	7.745471	0.130968
b	2.813037	3.321675	2.448558	5.716801	6.425568	4.094868
σ_b	15.05265	74124205	7.651339	6.069041	45225472	5.253221
Adj R^2	0.987981			0.974426		

Delboeuf, Difference of Gaussians, retinal distance*PSE

	Single-ring			*Two rings*		
	observed	*97.5%*	*2.5%*	*observed*	*97.5%*	*2.5%*
a	0.332216	0.419491	0.258008	0.290363	0.35234	0.24433
σ_a	0.150653	0.196236	0.113616	0.108989	0.16428	0.077422
b	0.282896	0.335524	-0.30969	0.51158	0.716816	-0.44942
σ_b	24.52702	79867555	5.902321	28.91758	4.68E+08	7.052637
Adj R^2	0.985185			0.956689		
